# Predictors of high tobacco use prevalence among adults in Autonomous Province of Vojvodina, Serbia

**DOI:** 10.1371/journal.pone.0320647

**Published:** 2025-04-25

**Authors:** Snežana Ukropina, Dragana Milijašević, Tanja Tomašević, Vesna Mijatović Jovanović, Dušan Čanković, Nataša Dragnić, Tatjana Tamaš, Vladimir Petrović

**Affiliations:** 1 Institute of Public Health of Vojvodina, Autonomous Province of Vojvodina, Novi Sad, Serbia; 2 Faculty of Medicine, University of Novi Sad, Autonomous Province of Vojvodina, Novi Sad, Serbia; 3 Oncology Institute of Vojvodina, Autonomous Province of Vojvodina, Novi Sad, Serbia; University of Modena and Reggio Emilia: Universita degli Studi di Modena e Reggio Emilia, ITALY

## Abstract

**Introduction:**

Introducing a sub-national surveillance system could significantly improve the monitoring of factors related to current tobacco use prevalence and the impacts of tobacco control measures. This study utilizes initial data from the newly established “**S**urveillance of **B**ehavioural **R**isk **F**actors of **N**on-**C**ommunicable **D**iseases in **V**ojvodina” (SBRF-NCD-V) system in the Autonomous Province of Vojvodina (APV), Serbia.

**Materials and methods:**

This cross-sectional study involved 3910 healthcare users aged 18 years and older, interviewed between May 20 and August 30, 2023, across all 44 Primary Healthcare Centers in the 7 districts of APV, with sampling proportional to Census data and stratified by gender, age and type of settlement. The questionnaire used was adapted from the BRFSS instrument developed by the CDC. Two multivariate binary logistic regression models analysed associations between current (daily or occasional) tobacco smoking and 6 sociodemographic factors, as well as 7 health-related or modifiable lifestyle risk factors, stratified by gender to calculate odds ratios.

**Results and discussion:**

The prevalence of daily (28.8%) or occasional (7.2%) tobacco smoking in APV (36.0%; 95% CI 34.5–37.5) surpasses national levels in Serbia and ranks among the highest in Europe. Dual use of combustible tobacco and e-cigarettes accounted for 21.7% of cases. Higher likelihoods of smoking were observed among age groups 18–34 compared to 35–49 years, rural residents (OR = 1.2; 95% CI = 41.4–46.7; p = 0.009), those with lower education levels (for primary school OR = 2.0; 95% CI = 11.7–15.4; p < 0.001), the employed (OR = 1.5; 95% CI = 64.5–69.5; p = 0.015), and individuals self-assessing their socioeconomic status as ‘poor or very poor’ (OR = 1.8; 95% CI = 11.0–14.6; p < 0.001). Significant predictors of smoking included poorer health self-assessment, higher self-assessed Body Mass Index, e-cigarette use, at least 1-hour daily tobacco exposure, alcohol consumption at least 3 times weekly and/or binge drinking, and lower physical activity levels.

**Conclusion:**

Factors contributing to the high prevalence of tobacco smoking in APV, Serbia, include younger age, rural residence, lower education, employment status, lower socioeconomic status, poorer health self-assessment, higher BMI (excluding women), e-cigarette use, tobacco exposure, alcohol consumption, and lower physical activity (excluding men). These findings underscore the need for targeted interventions, such as the efficacy of local inspection services and monitoring plans, to support policy measures aimed at addressing tobacco use in the region.

## Introduction

Global monitoring of tobacco use prevalence is an essential part of the MPOWER policy set of measures for control of the global tobacco epidemic. According to the World Health Organization (WHO) Framework Convention on Tobacco Control (FCTC), MPOWER stands for: “M – Monitor tobacco use and prevention policies; P - Protect people from tobacco smoke; O - Offer help to quit tobacco use; W - Warn about the dangers of tobacco; E - Enforce bans on tobacco advertising, promotion, and sponsorship; R - Raise taxes on tobacco”. Tobacco control is an integral part of the UN development agenda by 2030, contributing to Sustainable Development Goal 3 (“target 3.a calls for strengthening implementation of the WHO FCTC in all countries”). Reliable prevalence data are crucial for the evaluation of the impact of global, national and sub-national tobacco control interventions [1–3].

In addition to national-level actions, policymaking is also taking place at sub-national, provincial or district level [3]. Many research showed that there were significant hotspots and regional variations in smoking prevalence between districts in countries [4–10] and some even suggested that better data on the prevalence of tobacco smoking on the subnational level could contribute to the full unravelling of the reason for mortality convergence and divergence between districts [11].

Serbia ratified the WHO FCTC in 2006 and introduced 81 out of 134 measures, *i.e.,* measures provided in all articles in this Treaty except those in Articles 17 and 18. Also, Serbia is among the highest achieving countries considering monitoring the prevalence of tobacco use, according to the latest “WHO report on the global tobacco epidemic, 2023” and belongs to the countries that together count 5.6 billion people, 71% of the world population, protected by at least one MPOWER measure [12,13].

The average daily smoking rate among the population aged 15 and older in Serbia stands at 27.1%. This rate has remained relatively stable for females over the last two waves of the National Health Survey (NHS) in 2013 and 2019, at 26.0% and 25.0%, respectively. For males, the rate has been decreasing, from 32.6% in 2013 to 29.4% in 2019 [14,15]. In earlier NHS waves (2000 and 2006), the target population was aged 20 and over, with daily smoking rates among females at 26.1% and 22.6%, respectively, and among males at 40.6% and 30.7% [16]. According to the new WHO data, current tobacco use prevalence trends (daily and occasionally use) among people aged 15 years and older (not age-standardized), 2000–2030, in Serbia, considered years 2000, 2005, 2010, 2015, 2020, 2025 and 2030 are 43.3%, 41.5%, 39.9%, 38.3%, 37.1%, 36.0%, and 34.6% - respectively. The projections data about current tobacco use prevalence (daily and occasionally use), in the observed time-span, will decline for males by 14.6% (from 50.2% in 2000 to 35.6% in 2030), and for females will decline only for 3.2% (from 36.9% in 2000 to 33.7% in 2030) [17]. The percentage of people who smoke cigarettes, cigars or pipe tobacco daily or occasionally (at least 1 cigarette per month but less than daily if a person ever smoked 100 cigarettes in her life) among the population in Serbia (30.5%) is above the average in EU countries (23%) [18].

Those trends suggest that Serbia still has not passed the 3^rd^ phase of a “four-stage model of the cigarette epidemic”, proposed by Lopez et al. (the first version of the model in 1994 and updated in 2012) [19,20]. This model was proposed to communicate the long delay between the widespread uptake of cigarette smoking and smoking-attributed mortality (SAM). Stage 1 represents the very beginning of the smoking epidemic (low prevalence in both genders, low SAM). In stage 2, prevalence rates increase in both sexes, which are higher among males, with increasing SAM. Stage 3 is the period in which, although smoking prevalence is stable or decreasing (first for males), SAM is increasing rapidly to the maximum. In stage 4 smoking prevalence and, eventually, SAM decrease towards lower limits.

According to the newly proposed composite MPOWER Score by Husain et al. [21], Serbia belongs to countries with “High MPOWER – High Prevalence (HM-HP)” score. The countries are categorized in four initial conditions based on their tobacco control preparedness measured by MPOWER score and smoking burden measured by age-adjusted adult daily smoking prevalence: (1) High MPOWER – high prevalence (HM-HP), (2) High MPOWER – low prevalence (HM-LP), (3) Low MPOWER – high prevalence (LM-HP) and (4) Low MPOWER – low prevalence (LM-LP). A unit increase of the MPOWER Score was associated with a 0.39 and 0.50 percentage points decrease in adult daily smoking prevalence for HM-HP and HM-LP countries, respectively.

Through all four waves of NHS from 2000 to 2019 in Serbia, the smoking prevalence among the population aged 15+ (in 2013 and 2019) and the population 20 and over (in 2000 and 2006) was significantly higher than in other part of the country. At the same time, the prevalence of several other behavioural risk factors for non-communicable diseases was steadily higher in APV than in other regions of Serbia [14–16]. This situation revealed the need for the introduction in APV the new provincial-based behavioural risk factors surveillance system for chronic non-communicable diseases (NCDs) among the adult population. The data on current tobacco smoking used in this survey are the first data from a newly introduced sub-national (provincial) surveillance system for non-communicable diseases in Serbia [22].

Apart from the need to evaluate the prevalence of tobacco use in the accordance to WHO MPOWER policy set of measures for the implementation of WHO FCTC, numerous contemporary researches worldwide suggest the importance of explaining predicting factors of single or dual tobacco product use, such as socio-demographic factors as well as other health-related risk factors or comorbidities.

The objective of this study is to explore predictors of tobacco use prevalence among the adult population in APV, Serbia, according to the data from a newly established behavioural risk-factors surveillance system.

## Materials and methods

### Study design and data source

The study was performed as a cross-sectional study. Computer-Assisted Personal Interviews (CAPI) were conducted face-to-face, with trained interviewers using mobile phones connected to a computer program on the server. A modified version of the CDC’s (the Centers for Disease Control and Prevention, United States of America) BRFSS (Behavioral Risk Factor Surveillance System) questionnaire was used [23]. Modification of 2021 CDC’s BRFSS questionnaire includes translation into Serbian language 7 out of 15 core sections: 1 (Health Status), 4 (Exercise), 5 (Hypertension Awareness), 7 (Chronic Health Conditions), 9 (Demographics), 11 (Tobacco use), 12 (Alcohol Consumption), without using optional modules.

This research was conducted by the Institute of Public Health of Vojvodina (IPHV), APV, Serbia – as a part of the “Program Task of the Special Public Health Program for the Territory of APV in the 2023 year: Surveillance of behavioural risk factors for NCD’s in the adult population in APV (SBRF-NCD-V)”, according to the Decision of the Government of APV (“Official Gazette of the APV”, No. 54, December 22, 2022) [22].

The sample consisted of the adult noninstitutionalized population (18 years and older) of APV, who use health care in all of the 44 Primary Healthcare Centers (PHCs), across all 45 municipalities in APV. This population is covered by compulsory universal health care insurance (managed by the Republic Fund for Health Insurance) at the rate of 98% (86.3% of insured persons have a contract with general practitioners), which includes the non-working population whose health insurance contributions are financed from the central state budget [24,25].

Respondents were beneficiaries of the PHC services of general medicine, occupational medicine, gynaecology, polyvalent patronage (providing nurse health service to postpartum women, infants and family members with chronic diseases and disabilities, offering health education at homes and in PHCs) and the service for child health care (where respondents were parents accompanying children). Based on the data of the Republic of Serbia Institute of Statistics on the number and structure of the population of APV (according to the Census data, total population of N = 1.740.230), a sample of the adult population was created, which was stratified according to gender, age and type of settlement for each of 45 municipalities whose population is using service of 44 PHCs. A sample size of n = 3910 was created based on data on the prevalence of risk factors in this population, with a smoking prevalence of 35.5%, based on data on smoking prevalence of 35.5%, confirmed in 2019 NHS being considered for the final sampling frame, with a precision of 1.5% and an accuracy of 95%. According to the Census data, proportionally for each of 7 districts of APV and then for each of 45 municipalities (two steps of sampling), the number of surveyed individuals according to gender, age, and type of settlement (urban and rural) was previously planned by the IPHV (stratification). The survey was conducted over 3.5 months, from May 20 to August 30, 2023, until the stratified data plan was fulfilled. Exclusion criteria were persons with mental impairment and persons who in any phase refused interview.

Ethical aspects of surveillance (compliance with protocol, questionnaire, written informed consent of respondents, and methodological instructions for implementation of the survey with participants’ rights, ensuring that the study adheres to regulations, ethical guidelines, and regulatory standards) were considered by the Ethics Committee of the Institute of Public Health of Vojvodina (Decision No. 01–745/2 dated April 18, 2023). Each respondent signed the Informed Consent for participation in the survey and it is stored separately from the database. No personal data were collected. No minors were included. The survey was anonymous, participation was voluntary, with no incentives provided, and collected data were kept confidential on the IPHV server.

### Data control and data processing

Interviews were completed electronically (CAPI), by trained health professionals (physicians and nurses) in all 44 PHCs in APV, by using a mobile phone with personalized and authorized access to the software through a specially created software application accessed through the IPHV website. This approach enhanced data quality by minimizing data entry errors, validation checks (checking the average data entry period), and automated skip patterns which ensure that participants provide valid and consistent responses (values within a certain range, values with a required character, values without a certain character etc.). The control of the collected data was carried out at several levels: at the level of supervisors, coordinators and program managers. The primary database was transformed into a form suitable for processing in the statistical processing program SPSS (Statistical Package for Social Sciences), version 23. Data control in the database, modification of features and analysis were carried out by a physician, specialists in social medicine and a software engineer.

### Outcome variables

We considered that globally, the most relevant predictors of current tobacco use of cigarettes, cigars, and pipes among adults are: gender, age, education level, place of settlement, socioeconomic status, marital status, employment status, self-assessed health status, physical activity, alcohol use, biological health risk factors (nutritional status, hypertension) and different comorbidities [26–32]. In several new researches, the same groups of predictors were relevant for explaining reasons for using new types of tobacco products, within different population groups, additionally taking into account certain specific factors for explanations of use patterns, like dual use [33–36]. In the research protocol of our study, we created variables based on the 2021 BRFSS Data File [37] and 7 out of 15 core sections of the questionnaire [23]. The only variable considering the number of smoked cigarettes and exposure to tobacco smoke was added based on indicators used in the 2019 NHS of Serbia [15].

The following variables were created for the purposes of this survey:

- Dependent variables: “Current tobacco smokers” (yes; no) - respondents who smoked at least 100 cigarettes during their lifetime and currently smoke occasionally or daily; “Former tobacco smokers“ were considered respondents who smoked at least 100 cigarettes during their lifetime, but do not smoke at the moment of survey; “Dual tobacco users“– current tobacco smokers and regular or occasional e-cigarettes users.
- Demographic and socio-economic variables (factors included in Model 1 of multivariable analysis): 1) Gender (male; female); 2) Age (18–34; 35–64; 65+ years); 3) Place of settlement (urban or rural); 4) Marital status (married or living with partner; living alone – those who are divorced, separated, widowed or never married); 5) Education level (primary school or less – incomplete primary school or complete primary school; secondary school; university degree); 6) Employment status (employed or self-employed; unemployed; inactive – those who are students, stay-at-home or unable to work; retired); 7) Self-assessment of socioeconomic status (very good or good; average; poor or very poor).
- Health-related and lifestyle risk factors variables (factors included in Model 2 of multivariable analysis): 1) Health self-assessment (excellent or very good; good; average or poor); 2) Nutritional status (self-assessed) in Body Mass Index/BMI [kg/m2] (BMI<18.5; BMI 18.5–24.9; BMI 25.0–29.9; BMI**≥**30); 3) Use of e-cigarettes – daily or occasionally (yes; no); 4) Exposure to tobacco smoke in closed premises daily for 1 hour at least (yes; no) – house, work, public closed space, restaurants and others are considered as closed premises; 5) Alcohol use more than 2 days per week and/or binge drinking (5 or more drinks on one occasion for men and 4 or more drinks on one occasion for women) once per month or more frequently (yes; no); 6) Physical activity – physically active persons were those responding they had been active in the last month (excluding work) or engaged in activities such as running, muscle strengthening exercises, gardening or walking for exercise (yes; no); 8) Hypertension diagnosed by a health professional (yes; no).

### Statistical analysis

For statistical analysis of data, the descriptive and inferential statistics methods were used. Attributive data are presented through frequency distributions and relative numbers. Differences between observed characteristics were checked using non-parametric methods (χ^2^-test). Numerical data are presented through mean values and measures of variability. The associations of current tobacco smoking with two separate groups of independent variables (6 sociodemographic variables and 7 health-related and modifiable lifestyle risk factors) were examined by two multivariate binary logistic regression models, with gender-stratified odds ratios and with Hosmer and Lemeshow goodness of fit test.

Missing values, as well as answers “I don’t want to answer” or “I don’t know” were excluded from the analysis. All values with p < 0.05 were considered statistically significant. Variables were selected for the multivariable models on the results of previously applied univariate statistical tests. We included only those variables in the logistic regression models for which we found that there are significant differences in the univariate model. Multicollinearity among independent variables was tested by the Variance Inflation Factor (VIF) values. The values of VIF are in the range 1 < VIF < 5, specifying that the variables are moderately correlated to each other (Model 1: 1.01 < VIF < 1.78; Model 2: 1.02 < VIF < 1.27). The small values of VIF corresponding to the variables show that there is no problem of collinearity. Statistical data software SPSS (Statistical Package for Social Sciences), version 23, was used for all statistical analyses.

## Results

This cross-sectional survey included 3910 persons, aged 18 and over, who were beneficiaries of primary health care, in APV, Serbia, during 2023. The average age of the respondents was 49.2 years (SD = 17.5), with the youngest respondent being aged 18 and the oldest 93. Among the respondents, the female population was 51.8%, and the male population was 48.2%. The majority of respondents (60.2%) lived in urban settlements, and 39.8% lived in rural type of settlement. According to marital status, 55.5% of females and 59.1% of males stated that they lived in marriage. A share of 19.3% of the respondents stated that they had never married, 15.4% of females and 23.5% of males. In APV, the largest percentage of residents aged 18 and over have a secondary education (57.9%) and almost a third have education at university degree (29.1%). According to the self-assessment of socioeconomic status, the largest number of respondents indicated an “average” socioeconomic status (49.3%), followed by “good” (34.0%). At the time of the survey, more than half of the respondents (53.5%) were employed. Men were more often employed or self-employed (63.2%) than women (56.1%) (Table 1).

**Table 1:** Sociodemographic characteristics of respondents, according to gender, АPV, Serbia, 2023 (n = 3910).

Sociodemographic characteristics	Total 3910 (100)	Male 1885 (48.2)	Female 2025 (51.8)	p-value^*^
	n (%)	n (%)	n (%)	
Place of settlement; n = 3910				
Urban	2354 (60.2)	1110 (58.9)	1244 (61.4)	0.104
Rural	1556 (39.8)	775 (41.1)	781 (38.6)	
Age [years]	$\bar{X}$ = 49.2 (SD = 17.5)	$\bar{X}$ = 47.9 (SD = 17.4)	$\bar{X}$ = 50.5 (SD = 17.6)	**<0.001**
Age groups [years]; n = 3910				
18–34	947 (24.2)	487 (25.8)	460 (22.7)	**0.001**
35–49	1014 (25.9)	521 (27.6)	493 (24.3)	
50–64	1048 (26.8)	482 (25.6)	566 (28.0)	
65+	901 (23.1)	395 (21.0)	506 (25.0)	
Marital status; n = 3885				
Married	2224 (57.2)	1105 (59.1)	1119 (55.5)	** <0.001**
Divorced	287 (7.5)	118 (6.3)	169 (8.4)	
Widow/widower	394 (10.1)	96 (5.1)	298 (14.8)	
Separated	35 (0.9)	20 (1.1)	15 (0.7)	
Never married	750 (19.3)	440 (23.5)	310 (15.4)	
Living with a partner	195 (5.0)	91 (4.9)	104 (5.2)	
Education level; n = 3902				
Incomplete primary school	72 (1.9)	19 (1.0)	53 (2.6)	** <0.001**
Primary school	433 (11.1)	172 (9.1)	261 (12.9)	
Secondary school	2261 (57.9)	1148 (61.0)	1113 (55.1)	
University degree	1136 (29.1)	543 (28.9)	593 (29.4)	
Employment status; n = 3902				
Employed	2086 (53.5)	997 (53.0)	1089 (53.9)	** <0.001**
Self-employed	236 (6.0)	192 (10.2)	44 (2.2)	
Unemployed 1 year or more	179 (4.6)	107 (5.7)	72 (3.6)	
Unemployed less than 1 year	96 (2.5)	55 (2.9)	41 (2.0)	
Stay-at-home	168 (4.3)	3 (0.2)	165 (8.2)	
Student	163 (4.2)	86 (4.6)	77 (3.7)	
Retired	923 (23.6)	415 (22.1)	508 (25.1)	
Unable to work	51 (1.3)	25 (1.3)	26 (1.3)	
Self-assessment of socioeconomic status; n = 3873				
Very good	254 (6.6)	141 (7.6)	113 (5.6)	0.062
Good	1318 (34.0)	654 (35.0)	664 (33.1)	
Average	1908 (49.3)	890 (47.7)	1018 (50.7)	
Poor	349 (9.0)	161 (8.6)	188 (9.5)	
Very poor	44 (1.1)	21 (1.1)	23 (1.1)	

**Fig 1:**
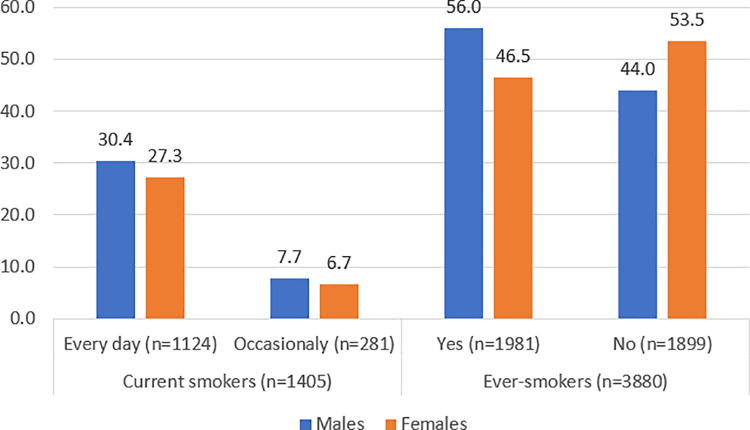
The proportion [%] of current and ever-smokers, APV, Serbia, 2023.

*χ2 test for all variables, except for age (Student t-test);

In APV in 2023, 28.8% of respondents consumed cigarettes daily (30.4% of males and 27.3% of females), and 7.2% occasionally (7.7% of males and 6.7% of females). When asked if they had consumed at least 100 cigarettes during their lifetime, slightly more than half of the respondents (51.1%) answered “yes” (9.5% males more than females) (Fig 1).

The prevalence of current smoking in the surveyed population was 36.0% (95%CI 34.5–37.5). A higher prevalence was recorded in males, compared to females (38.2% vs. 33.9%) (p = 0.006).

The highest percentage of smokers in the observed population belonged to the age category 35–49 and 50–64, while the lowest percentage was recorded in the category 65 and over (41.8%; 41.6%; 23.7%, respectively) (p < 0.001). In our analysis, a statistically significantly higher percentage of smokers was found in rural areas, compared to urban areas (39.8% vs. 33.4%) (p < 0.001).

Concerning marital status, the highest percentage of smokers was in the divorced category (47.0%), and the lowest was in the widow/widower category (28.6%) (p < 0.001).

The prevalence of smoking is the highest among persons with secondary education (39.7%), while it is the lowest in the category of university degree education (28.1%) and this difference is statistically significant (p < 0.001). The highest percentage of smokers (42.9%) was found among the unemployed population, while the percentage was the lowest in the category of students (20.9%). The highest percentage of smokers was recorded among respondents with the worst financial condition (45.2%) (p < 0.001) (Table 2).

**Table 2:** Prevalence of current tobacco smoking in the surveyed population in APV in relation to the socio-demographic characteristics of the respondents, 2023.

Variable	Current tobacco smoking (daily or occasionally)					Current tobacco smoking (daily or occasionally)					p-value*
	Mail				p-value		Female				p-value
	Yes		No		Yes		No				
	n(%)	95% CI	n(%)	95% CI	n(%)	95% CI	n(%)	95% CI			
**Age groups (years**); n = 3905											
18–34	183(37.6)	33.3–42.0	304(62.4)	58.0–66.7	<0.001	150(32.6)	28.3–37.1	310(67.4)	62.9–71.7	<0.001	<0.001
35–49	214(41.1)	36.8–45.4	307(58.9)	54.6–63.2		209(42.6)	38.1–47.1	282(57.4)	52.9–61.9		
50–64	220(45.6)	41.1–50.2	262(54.4)	49.8–58.9		216(38.2)	34.2–42.4	349(61.8)	57.6–65.8		
65+	102(25.9)	21.6–30.5	292(74.1)	69.5–78.4		111(22.0)	18.4–25.8	394(78.0)	74.2–81.6		
**Place of settlement**; n = 3905											
Urban	387(34.9)	32.1–37.8	722(65.1)	62.2–67.9	<0.001	399(32.1)	29.5–34.8	843(67.9)	65.2–70.5	0.029	<0.001
Rural	332(42.8)	39.3–46.4	443(57.2)	53.6–60.7		287(36.8)	33.4–40.3	492(63.2)	59.7–66.6		
**Marital status**; n = 3880											
Married or living with a partner	472(39.5)	36.7–42.3	724(60.5)	62.5–65.5	0.004	424(34.7)	32.1–37.5	797(65.3)	62.5–67.9	<0.001	<0.001
Divorced or separated	66(47.8)	39.3–56.5	72(52.2)	43.5–60.7		85(46.4)	39.1–54.0	98(53.6)	46.0–60.9		
Widow/ widower	30(31.6)	22.4–41.9	65(68.4)	58.1–77.6		82(27.6)	22.6–33.1	215(72.4)	66.9–77.4		
Never married	145(33.0)	28.6–37.6	295(67.0)	62.4–71.4		91(29.4)	24.3–34.8	219(70.6)	65.2–75.7		
**Education level**; n = 3897											
Primary school or less	86(45.0)	37.8–52.4	105(55.0)	47.6–62.2	<0.001	103(32.9)	27.7–38.4	210(67.1)	61.6–72.3	0.001	<0.001
Secondary school	483(42.1)	39.2–45.0	665(57.9)	55.0–60.8		414(37.2)	34.4–40.1	698(62.8)	59.9–65.6		
University degree	150(27.7)	23.9–31.6	392(72.3)	68.4–76.1		168(28.4)	24.8–32.2	423(71.6)	67.8–75.2		
**Employment status**; n = 3897											
Employed or self-employed	504(42.4)	39.6–45.3	685(57.6)	54.7–60.4	<0.001	434(38.3)	35.5–41.2	698(61.7)	58.8–64.5	<0.001	<0.001
Un-employed	69(42.6)	34.9–50.6	93(57.4)	49.4–65.1		49(43.4)	34.1–53.0	64(56.6)	47.0–65.9		
Stay-at-home	2(66.7)	–	1(33.3)	–		55(33.7)	26.5–41.6	108(66.3)	58.4–73.5		
Student	22(25.6)	16.8–36.1	64(74.4)	63.9–83.2		12(15.6)	8.3–25.6	65(84.4)	74.4–91.7		
Retired	107(25.8)	21.7–30.3	307(74.2)	69.7–78.3		127(25.0)	21.3–29.1	380(75.0)	70.9–78.7		
Unable to work	11(44.0)	24.4–65.1	14(56.2)	34.9–75.6		7(26.9)	11.6–47.8	19(73.1)	52.2–88.4		
**Self-assessment of socioeconomic status**; n = 3868											
Very good or good	266(33.5)	30.2–36.9	529(66.5)	63.1–69.8	<0.001	243(31.3)	28.1–34.7	533(68.7)	65.3–71.9	0.036	<0.001
Average	354(39.8)	36.6–43.1	535(60.2)	56.9–63.4		353(34.7)	31.8–37.8	663(65.3)	62.2–68.2		
Poor or very poor	92(50.5)	43.1–58.0	90(49.5)	42.0–56.9		85(40.5)	33.8–47.4	125(59.5)	52.6–66.2		

*χ^2^ test for total

**Fig 2:**
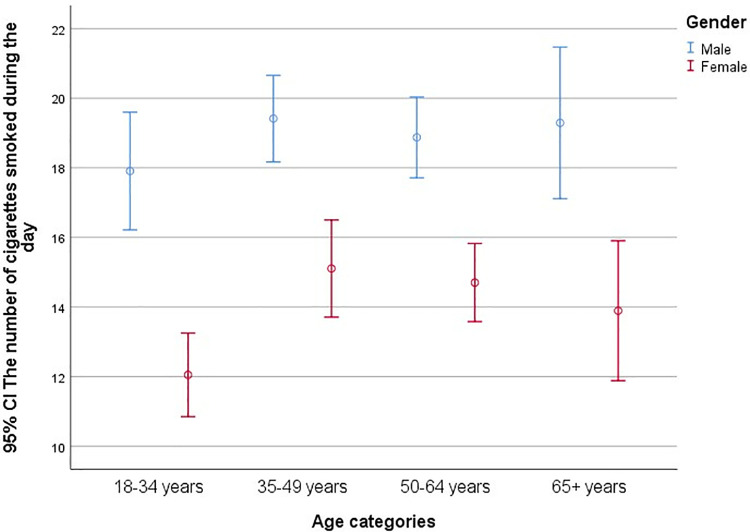
The average number of cigarettes per day [95% CI], according to gender and age, APV, Serbia 2023.

Among 1081 respondents who smoke daily (43 respondents did not answer), the average number of cigarettes smoked per day was 17.7 and 53.0% of smokers consumed one or more packs per day ($\bar{X}$  = 17.7; Min = 1; Max = 100; SD = 8.7), with the highest mean value among males ($\bar{X}$  = 19.14; SD = 8.4) and females ($\bar{X}$  = 15.1; SD = 9.6) aged 35–49 years (Fig 2).

**Fig 3:**
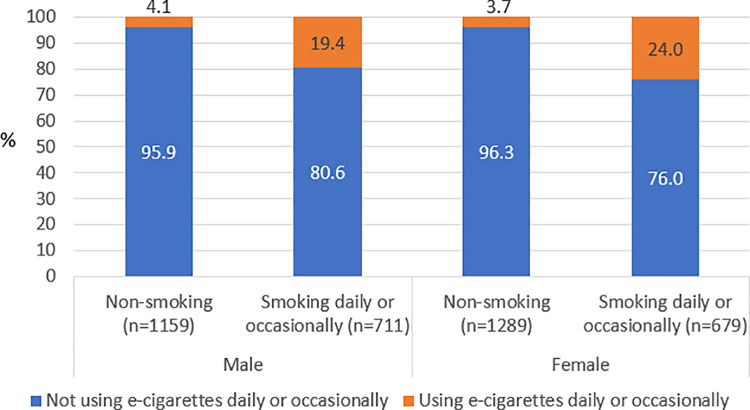
Proportion of e-cigarettes users [%], according to gender and smoking status, APV, Serbia 2023.

Among 1390 respondents out of 1405 who smoke daily or occasionally and answered on question about e-cigarettes use (daily or occasionally), 21.7% belongs to dual-users (19.4% males and 24.0% females). Among non-smokers, 4.1% of males and 3.7% of females are using e-cigarettes (Fig 3).

Respondents aged 18–34 years were 1.7 times more likely to be daily cigarette/combustible tobacco product users (OR = 1.7; 95% CI = 1.2–2.5; p = 0.003) than those aged 65 and older, and those aged 35–64 years were twice as likely (OR = 2.0; 95% CI = 1.5–2.7; p < 0.001). Considering gender-stratified odds ratios, this was relevant just for female respondents in both age groups.

With regard to place of settlement, survey participants from rural areas were 1.2 more likely to smoke daily or occasionally than those who lived in urban areas (OR = 1.2; 95% CI = 1.1–1.4; p = 0.009).

The respondents with less education level accomplished were more likely to be daily or occasional smokers – those with primary school or less – twice time more likely (OR = 2.0; 95% CI = 1.6–2.6; p < 0.001), and 1.7 times more likely if they finished secondary school (OR = 1.7; 95% CI = 1.4–2.0; p < 0.001), both compared with participants who finished university degree education level. Gender-stratified odds ratios showed the same relations, with some higher influence of the same pattern among males than females because they had higher odds of being a smoker if they had a lower education degree.

Employed respondents had higher odds for daily or occasional smoking (OR = 1.5; 95% CI = 1.1–1.9; p = 0.015) than those who are in pension, especially if they are male (OR = 2.0; 95% CI = 1.3–3.1; p = 0.004), with no significance in female-related odds ratios.

The survey participants who self-assessed their socioeconomic status as “poor or very poor” were 1.8 times more likely to be daily or occasional smokers (OR = 1.8; 95% CI = 1.4–2.3; p < 0.001). The higher odds were found among males (OR = 1.9; 95% CI = 1.3–2.7; p < 0.001) than females (OR = 1.7; 95% CI = 1.2–2.4; p = 0.003) (Table 3).

**Table 3:** Current tobacco smoking daily or occasionally (n = 3905) according to the sociodemographic factors (Model 1), proportions (in % and 95%CI) and multivariate binary logistic regression (gender-stratified odds ratios).

Predictor variables Model 1	Female			Male			Total		
	% (95% CI)	OR (95% CI)	*p*	% (95% CI)	OR (95% CI)	*p*	% (95% CI)	OR (95% CI)	*p*
**Age** (n = 3905)									
18–34 year	21.8 (18.8–25.1)	2 (1.3–3.2)	**0.004**	25.5 (22.3–28.8)	1.3 (0.8–2.2)	0.333	23.7 (21.5–26.0)	1.7 (1.2–2.5)	**0.003**
35–64 year	62.0 (58.2–65.6)	2.6 (1.7–3.9)	** < 0.001**	60.3 (56.7–64.0)	1.4 (0.9–2.3)	0.132	61.1 (58.5–63.7)	2.0 (1.5–2.7)	** < 0.001**
65+ year	16.2 (13.5–19.2)	Ref.	–	14.2 (11.7–17.0)	Ref.	–	15.2 (13.3–17.1)	Ref.	–
**Place of settlement** (n = 3905)									
Urban	58.2 (54.4–61.9)	Ref.	–	53.8 (50.1–57.5)	Ref.	–	55.9 (53.3–58.6)	Ref.	–
Rural	41.8 (38.1–45.6)	1.2 (1.0–1.5)	0..071	45.2 (42.5–49.9)	1.2 (1.0-1.5)	0.072	44.1 (41.4-46.7)	1.2 (1.1-1.4)	**0.009**
**Marital status** (n = 3880)									
Married/living with a partner	62.2 (58.4–65.8)	0.9 (0.7–1.1)	0.448	66.2 (62.8–69.7)	1.2 (0.9–1.5)	0.197	64.2 (61.7–66.7)	1.0 (0.9–1.2)	0.704
Living alone	37.8 (34.2–41.6)	Ref.	–	33.8 (30.3–37.4)	Ref.	–	35.8 (33.3–38.3)	Ref.	–
**Education level** (n = 3897)									
Primary school or less	15.0 (12.4–17.9)	1.7 (1.2–2.5)	**0.003**	12.0 (9.7–14.6)	2.5 (1.7–3.7)	** < 0.001**	13.5 (11.7–15.4)	2.0 (1.6–2.6)	** < 0.001**
Secondary school	60.4 (56.7–64.1)	1.5 (1.2–1.9)	** < 0.001**	67.2 (63.6–70.6)	1.8 (1.4–2.3)	** < 0.001**	63.9 (61.3–66.4)	1.7 (1.4–2.0)	** < 0.001**
University degree	24.5 (21.3–27.9)	Ref.	–	20.9 (17.9–24.0)	Ref.	–	22.6 (20.5–24.9)	Ref.	–
**Employment status** (n = 3897)									
Employed or self-employed	63.5 (59.7–67.1)	1.2 (0.8–1.8)	0.464	70.5 (67.0–73.8)	2.0 (1.3–3.1)	**0.004**	67.0 (64.5–69.5)	1.5 (1.1–1.9)	**0.015**
Unemployed	7.2 (5.3–9.4)	1.3 (0.7–2.2)	0.423	9.7 (7.6–12.1)	1.5 (0.9–2.6)	0.126	8.4 (7.0–10.0)	1.3 (0.9–1.9)	0.171
Inactive	9.8 (7.7–12.3)	0.7 (0.4–1.0)	0.071	3.4 (2.2–5.0)	1.1 (0.5–2.1)	0.847	6.5 (5.3–7.9)	0.8 (0.5–1.1)	0.124
In pension	19.6 (16.7–22.8)	Ref.	–	16.5 (13.9–19.4)	Ref.	–	18.0 (16.0–20.1)	Ref.	–
**Self-assessment of socioeconomic status** (n = 3868)									
Very good or good	35.7 (32.1–39.4)	Ref.	–	37.4 (33.8–41.0)	Ref.	–	36.5 (34.0–39.1)	Ref.	–
Average	51.8 (48.0–55.6)	1.1 (0.9–1.4)	0.220	49.7 (46.0–53.5)	1.2 (1.0–1.5)	0.091	50.8 (48.1–53.4)	1.2 (1.0–1.4)	0.033
Poor or very poor	12.5 (10.1–15.2)	1.7 (1.2–2.4)	**0.003**	12.9 (10.5–15.6)	1.9 (1.3–2.7)	** < 0.001**	12.7 (11.0–14.6)	1.8 (1.4–2.3)	** < 0.001**

OR – Odds Ratio; 95% CI – 95% Confidence Interval; Ref – Reference value.

The respondents who self-assessed their health as “good” (OR = 1.5; 95% CI = 1.2–1.9; p = 0.001) or “average or poor” (OR = 1.6; 95% CI = 1.2–2.0; p = 0.001) were about 1.5 times more likely to smoke daily or occasionally and this was relevant for both genders.

Survey participants who self-assessed (according to the BMI) having normal weight, with BMI 18.5–24.9 (OR = 1.3; 95% CI = 1.0–1.7; p = 0.046) and overweight, with BMI 25.0–29.9 (OR = 1.3; 95% CI = 1.0–1.7; p = 0.034), were more likely to have been daily or occasionally smokers in comparison with those who were obese (BMI ≥ 30). Gender-stratified odds ratios showed that female respondents who have been underweight were almost 2.5 times more likely to daily or occasionally smoke cigarettes (OR = 2.4; 95% CI = 1.0–5.6; p = 0.042) compared with female participants who were obese.

Respondents who used e-cigarettes daily or occasionally were almost 8 times more likely to be a daily or occasionally cigarette smokers (OR = 7.9; 95% CI = 5.7–11.0; p < 0.001), with the almost same gender-stratified odds between female (OR = 7.8; 95% CI = 5.0–12.7; p < 0.001) and male (OR = 8.2; 95% CI = 5.1–13.3; p < 0.001).

Interviewed individuals who reported that they were exposed to tobacco smoke in closed premises daily for 1 hour at least, had almost 11 times higher odds of being daily or occasionally smokers than those with shorter exposure to environmental tobacco smoke (OR = 10.9; 95% CI = 8.9–13.3; p < 0.001), with almost the same gender-stratified odds between female (OR = 10.2; 95% CI = 7.7–13.4; p < 0.001) and male (OR = 11.5; 95% CI = 8.6–15.4; p < 0.001).

Respondents who, in the last month, were not physically active (excluding work) were 1.4 times more likely to smoke daily or occasionally than those who were physically active (OR = 1.4; 95% CI = 1.1–1.7; p = 0.004) and gender-stratified odds showed significance among women (OR = 1.4; 95% CI = 1.0–1.9; p = 0.028).

Participants who were alcohol users more than 2 days per week and/or binge drinking once per month or more frequently, were 1.7 times more likely to smoke daily or occasionally than those without that type of risk (OR = 1.7; 95% CI = 1.4–2.2; p < 0.001) and that was related to gender-stratification too, with almost the same level of odds among females (OR = 1.7; 95% CI = 1.1–2.6; p = 0.017) and males (OR = 1.8; 95% CI = 1.3–2.3; p < 0.001)

Diagnosed hypertension by a health professional does not appear as a significant predictor for daily or occasionally current cigarette smoking (Table 4).

**Table 4:** Current tobacco smoking (daily or occasionally) according to the health-related or modifiable lifestyle risk factors (Model 2), proportions (in % and 95%CI) and multivariate binary logistic regression (gender-stratified odds ratios).

Predictor variables Model 2	Female			Male			Total		
	% (95% CI)	OR (95% CI)	*p*	% (95% CI)	OR (95% CI)	*p*	% (95% CI)	OR (95% CI)	*p*
**Health self-assessment** (n = 3876)									
Excellent or very good	22.0 (18.9–25.3)	Ref.	–	27.1 (23.8–30.5)	Ref.	–	24.6 (22.3–26.8)	Ref.	–
Good	37.9 (34.2–41.7)	1.4 (1.0–2.0)	**0.044**	38.9 (35.3–42.6)	1.6 (1.1–2.2)	**0.011**	38.4 (35.9–41.0)	1.5 (1.2–1.9)	**0.001**
Average or poor	40.1 (36.4–43.9)	1.4 (1.0–2.1)	**0.046**	34.0 (30.5–37.6)	1.8 (1.2–2.6)	**0.004**	37.0 (34.4–39.6)	1.6 (1.2–2.0)	**0.001**
**Nutritional status (self-assessed) in BMI** [kg/m2] (n = 3662)									
BMI<18.5	3.7 (2.4–5.5)	2.4 (1.0–5.6)	**0.042**	0.4 (0.1–1.3)	0.6 (0.9–3.9)	0.569	2.1 (1.4–3.0)	1.8 (0.9–3.7)	0.128
BMI 18.5––24.9	52.5 (48.5–56.4)	1.4 (0.9–2.0)	0.097	36.8 (33.1–40.6)	1.3 (0.9–1.9)	0.225	44.5 (41.8–47.2)	1.3 (1.0–1.7)	**0.046**
BMI 25.0––29.9	31.0 (27.4–34.7)	1.4 (1.0–2.1)	0.082	43.8 (40.0–47.7)	1.3 (0.9–1.8)	0.195	37.5 (34.9–40.2)	1.3 (1.0–1.7)	**0.034**
BMI≥30	12.8 (10.3–15.6)	Ref.	–	19.0 (16.1–22.2)	Ref.	–	15.9 (14.0–18.0)	Ref.	–
**Use of e-cigarettes (daily or occasionally)** (n = 3876)									
Yes	24.0 (20.8–27.4)	7.8 (5.0–12.7)	** <0.001**	19.4 (16.6–22.5)	8.2 (5.1–13.3)	** <0.001**	21.7 (19.5–23.9)	7.9 (5.7–11.0)	** <0.001**
No	76.0 (72.6–79.2)	Ref.	–	80.6 (77.5–83.4)	Ref.	–	78.3 (76.1–80.5)	Ref.	–
**Exposure to tobacco smoke in closed premises daily for 1 hour at least** (n = 3843)									
Yes	56.6 (52.8–60.4)	10.2(7.7–13.4)	** <0.001**	60.6 (56.9–64.2)	11.5(8.6–15.4)	** <0.001**	58.7 (56.0–61.3)	10.9(8.9–13.3)	** <0.001**
No	43.4 (39.6–47.2)	Ref.	–	39.4 (35.8–43.1)	Ref.	–	41.3 (38.7–44.0)	Ref.	–
**Alcohol use more than 2 days per week and/or binge drinking** ≥**1 per month** (n = 3289)									
Yes	14.1 (11.4–17.2)	1.7 (1.1–2.6)	**0.017**	50.2 (46.0–54.4)	1.8 (1.3–2.3)	** <0.001**	31.9 (29.2–34.7)	1.7 (1.4–2.2)	** <0.001**
No	85.9 (82.8–88.6)	Ref.	–	49.8 (45.6–54.0)	Ref.	–	68.1 (65.3–70.8)	Ref.	–
**Physical activity** (n = 3863)									
Yes	72.0 (68.5–75.4)	Ref.	–	75.5 (72.1–78.6)	Ref.	–	73.8 (71.4–76.1)	Ref.	–
No	28.0 (24.6–31.5)	1.4 (1.0–1.9)	**0.028**	24.5 (21.4–27.9)	1.4 (1.0–2.0)	0.053	26.2 (23.9–28.6)	1.4 (1.1–1.7)	**0.004**
**Hypertension (diagnosed by a health professional)** (n = 3804)									
Yes	45.7 (41.9–49.6)	1.1 (0.8–1.4)	0.686	46.1 (42.4–49.9)	0.9 (0.7–1.2)	0.439	45.9 (43.3–48.6)	1.0 (0.8–1.2)	0.933
No	54.3 (50.4–58.1)	Ref.	–	53.9 (50.1–57.6)	Ref.	–	54.1 (51.4–56.7)	Ref.	–

OR – Odds Ratio; 95% CI – 95% Confidence Interval; Ref – Reference value.

## Discussion

This study used data about the prevalence of current tobacco smoking based on the newly introduced behavioural risk factors surveillance system for non-communicable diseases for the Autonomous Province of Vojvodina (APV), Serbia (“SBRF-NCD-V”).

The determined prevalence of daily or occasional current tobacco smoking of cigarettes among the adult population (aged 18 and over) in APV was 36.0% (95% CI 34.5–37.5) – 28.8% of the respondents smoked tobacco products daily, and 7.2% occasionally. These results showed a higher prevalence of current tobacco smoking in comparison with the latest national smoking prevalence results for the population aged 15 and over, in Serbia, in 2019 (30,5% prevalence of daily and occasionally smokers of cigarettes, cigars or pipe), which can be explained due to the methodology differences (our study is not а national representative household survey but collected data from people aged 18+ who use health care in 44 PHC’s in all of 45 municipalities in APV) or influence of multiple other related factors [15].

Statistical analyses of current tobacco use should be stratified by gender for several important reasons: identification of important gender differences in current tobacco usage patterns in terms of frequency, types of tobacco product used, and the number of cigarettes; certain demographic, socioeconomic, or behavioural risk factors are associated with tobacco use differently for men and women and some social factors can change; gender stratification can reveal disparities in tobacco use that may be linked to broader health inequalities, enabling targeted interventions to reduce these disparities; analysing data separately can highlight these differences and help shape more effective tobacco control strategies [38,39].

In multivariable Model 1, the same predictors variables of higher prevalence of daily or occasional current tobacco smoking were confirmed like in univariate statistical analysis (males, aged 35–64, rural settlement, lower education attainment, unemployment and lower socioeconomic status), except marital status. Gender-stratified odds were not relevant for marital and employment status. These findings are corresponding with other researches and reports [40,41,42].

In comparison to the current tobacco smoking prevalence of populations in other national, sub-national levels or regions, the prevalence of 36.0% (38.2% among males and 33.9% among females) should be considered as high, especially among women, because globally, the average smoking prevalence is about 20% [43].

Newly findings from nationally representative survey data from 82 countries, found that the average smoking prevalence was 16.5% and varies significantly between countries, from 1.1% in Ghana to 50.6% in Kiribati [44]. Although variation was wide between countries and by tobacco product (smoking and smokeless tobacco), for many low- and middle- income countries (LMIC), certain demographic groups were found to have higher rates of tobacco use, including men, individuals with lower education and household wealth, those living in rural areas, and higher age. The results of our study are aligned with a study by Theilmann M. et al. [44], considering sociodemographic disparities in smoking prevalence. The estimates on tobacco smoking from a large cross-sectional survey in Europe, Gallus S. et al. [45], concluded that lower socio-economic status is a major determinant of smoking habit in both sexes.

Rural sub-national areas are often predictors of a higher probability of smoking. For example, an Australian study of Das S et al. [46] found a positive association between remoteness and indigeneity (more prominent in the Northern Territory) with a higher prevalence of smoking patterns.

The survey from Indonesia, which investigated disparities in tobacco smoking prevalence in 514 districts, found a huge disparity in smoking prevalence between districts, between 9 and 81% for males and 0–41% for females, with significantly higher smoking prevalence among men in poorest districts and higher smoking prevalence among women in districts with less educated population [4]. In our study, according to the gender-stratified odds ratios, we didn’t find disparities between men and women according to the socioeconomic status and education attainment. Both, women and men, were more likely to be smokers if they self-assessed socioeconomic status as poor or very poor and had lower education.

Marital status in our study didn’t appear as a significant predictor of higher current tobacco smoking, although in some researches divorced status showed a significant association with the likelihood of current cigarette smoking [47,48]. These inconsistencies of results with our research could be explained due to the methodology differences, because in our study we combined a group of divorced individuals with those who were never married or separated or widowed, designated as “living alone”.

Influence of different factors of socioeconomic development was confirmed by several other studies with focus on sub-national smoking prevalence assessment. One study in Russia found that increased Industrial index in the regions is associated with the individual probability of smoking [9] and other study between Virginia Counties (U.S.A.) discovered that higher social vulnerability index is associated with increased cigarette use [10].

Poorer self-assessed health status in our study appeared as predictor of higher odds for smoking prevalence, as well as in some other studies in the United Kingdom [49], India [8] and Poland [50].

Considering modifiable lifestyle risk factors as predictors of daily or occasionally tobacco smoking, most studies confirm steadily increased probability with a higher prevalence of these health risk factors, with insight into new phenomena such as “dual use of tobacco products” (dual use of factory-made combustible cigarettes, other tobacco products and electronic cigarettes) or “triple burden“ of smoking, smokeless tobacco and alcohol consumption [8,51].

The use of e-cigarettes, whether daily or occasionally, was examined in this survey as a predictor of daily or occasional smoking of cigarettes. Other studies that have researched patterns and predictors of dual use of tobacco products have also explored how tobacco use predicts e-cigarette consumption, particularly in terms of cessation attempts [52]. In our survey, the use of e-cigarettes emerged as a strong predictor of tobacco smoking, which aligns with the findings of another study conducted by Owusu D et al. [53]. Furthermore, considering that there is global evidence showing that the current prevalence of e-cigarette vaping among individuals who have previously smoked conventional cigarettes is 39%, our results are consistent with this finding [54]. Focusing on daily and occasional use of e-cigarettes allows for a clearer understanding of different usage patterns of cigarette use also, because there is evidence that the relationship between e-cigarette use and current cigarette smoking is attributable to shared risk factors. These categories can help distinguish between habitual and experimental use and dual use. While cross-sectional studies can identify associations between e-cigarette and cigarette use, they cannot definitively establish causal relationships or the directionality of use (e.g., whether e-cigarettes lead to cigarette smoking or vice versa). One survey among adolescents revealed the possibility that “the gateway works in two directions, that e-cigarette and tobacco use share common risk factors, or that both mechanisms apply” [55,56,57].

Exposure to secondhand smoke (SHS) in our study contributes to the prevalence of tobacco use with 11- time higher odds compared with those respondents who are not exposed to tobacco smoke for at least 1 hour daily. In one study in Greece, tobacco ever smokers reported higher percentages of passive smoking either overall or in public and working places, compared to never smokers [58]. In the Portugal study from Alves R.F. et al. [59] exposure to SHS also contributes to the prevalence of tobacco use. Those findings can be explained with evidence that SHS exposure at home, either for smokers or non-smokers, was strongly associated with living with a smoker and it is present in a multicultural context [60,61].

Regarding the alcohol consumption as predictor of tobacco smoking, results from our study are similar to other studies that found higher odds of daily or occasionally smoking in those who do not drink moderately, regardless of sex. Findings substantially report higher prevalence of any form of tobacco and alcohol consumption (per week and/or binge drinking) among men in comparison to women [8,62], even in sub-national level [63] and regardless of some other socioeconomic aspects like unemployment [64].

Although BMI is not typically a causal or direct risk factor for smoking, we include this variable in the second explanatory model because we want to explore the potential association between BMI and smoking prevalence. This could confound the association between smoking and other health outcomes, so including several health outcomes helps isolate their respective impacts. Our study reveals that a lower BMI is a predictor of higher current smoking prevalence when comparing overweight respondents as the reference group. The gender-stratified model indicates that underweight women have greater odds of being current or occasional smokers compared to overweight women. The association between smoking and lower BMI is further supported by pooled data from 21 twin cohorts in a cross-sectional study by Piirtola M. et al. [65]. This study, *inter alia*, analyzed monozygotic twin pairs using linear fixed-effects regression models over 10-year periods, revealing that the smoking co-twin had an average BMI that was 0.57 kg/m^2^ lower in men and 0.65 kg/m^2^ lower in women compared to the never-smoking co-twin. In other type of study design, the analysis of lifetime smoking by Taylor E.A. et al. [66], provided clear evidence for a causal effect of BMI on increased lifetime smoking behaviour and also found some evidence for a causal effect of higher BMI on smoking heaviness within smokers.

In our research, respondents with insufficient physical activity had higher odds of being current tobacco smokers than those who were physically active, regardless the sex. From one of the first systematic reviews in this century about smoking and physical activity [67] to the recent studies which researched influence of COVID-19 on main lifestyle factors, physical activity is negatively associated with smoking, especially among women [68]. Data are less consistent considering man and they depend on how physical activity is defined (any or moderate exercise; total minutes of physical activity etc.).

The fact that we did not find statistically significant difference in odds of current tobacco smokers prevalence between those who had and had not hypertension (diagnosed by a health professional), although smoking is claimed to be a cause of hypertension [69], can be explained by low awareness of need for smoking cessation among respondents who had hypertension. Another possible explanation, according to the recent study designed by Ninomiya Y. et al. [70], could be that among those with hypertension exist a significant group of former smokers, who gained more weight through the cessation period, which led to blood pressure elevation.

The study which included 3431 surveys by Dai X et al. [71] showed that although smoking prevalence has declined by 27.2% for men since 1990, and by 37.9% for women, smoking is still common and causes a significant health burden worldwide, with unequal declines between countries, shifting the epidemic progressively to LMIC.

Given that the Global Study on the Burden of Disease identified smoking as the second most common risk factor for premature mortality and disability in Serbia, it is crucial to monitor prevalence data at a sub-national level [72].

According to the WHO FCTC, the most cost-effective tobacco control measures on the highest level of achievement rely on the introduction of national policy and their progress: a ≥75% tax share in cigarette prices; complete smoke-free public spaces; an age limit above 18 for tobacco sales; a ban on all advertising, promotion, and sponsorship; a national education campaign with at least seven key features, including TV/radio ads; large health warnings on cigarette packages; and national cessation programs, including quitlines and coverage for nicotine-replacement therapy and other services [12].

Tobacco control measures introduced at the national level often include subnational or local monitoring plans, and the effectiveness of implementing the WHO FCTC depends on how well laws and regulations are enforced at these subnational levels (for example the efficacy of local inspection services, local public health plans protected from the commercial and other vested interests of the tobacco industry etc.). Constant monitoring of tobacco use on subnational levels could contribute to tailoring nationally introduced tobacco control measures, or even enhance the scope of their achievement.

### Limitations of the study

The prevalence estimated in this study, which focused on primary healthcare users, may not accurately represent the prevalence of APV in the overall population. This is despite the high rate of compulsory insurance and the sampling proportional to Census data, which could affect the generalizability and comparability of the results.

Given that individuals who utilize health services may differ from the general population in factors such as age, gender, and morbidity, and considering that tobacco use is associated with a variety of health issues, the prevalence observed in this study—conducted among primary health care users—may not accurately reflect the general population prevalence of APV. This is particularly relevant when compared to household-based population studies, which were not feasible due to resource constraints. To address this limitation, three strategies were employed: (1) respondents were primary healthcare users of all healthcare services across all 44 PHCs in each of 45 municipalities in APV, considering that Serbia’s health system is characterized by universal health coverage, ensuring that more than 80% of the population is enrolled with general practitioners and has access to preventive health services; (2) the sampling process was stratified based on Census data regarding gender, age, and settlement type across two phases (districts and municipalities), which helped align the sample structure as closely as possible with more accurate, but less cost-effective, household-based methods; and (3) establishing “SBRF-NCD-V” ensures frame for longitudinal monitor of risk factors for NCD’s and comparing results with tobacco control measures.

The variable construction according to the 2021 BRFSS protocol requires standardization in the near future for the Serbian population. Some dilemmas could not be resolved in cross-sectional study design. The answer to the questions about the causes and ways of reducing the prevalence of smoking can be determined in longitudinal studies and qualitative research, as well as evaluations of health promotion measures.

## Conclusions

The findings of this study presented the first data from the newly behavioural risk-factors surveillance system on the sub-national level, in the Autonomous Province of Vojvodina, Serbia (SBRF-NCD-V). The results suggest that the prevalence of current tobacco smoking (36.0%; 95%CI 34.5–37.5) is higher than on the national level and among the highest in Europe. Sociodemographic predictors of higher prevalence of current tobacco smoking are: younger age, rural place of settlement, lower education level, employment and lower self-assessed socioeconomic status. Health-related and lifestyle risk factors associated with a higher prevalence of current tobacco smoking are: lower health self-assessment, higher self-assessed BMI (except among women), use of e-cigarettes, exposure to tobacco smoke, alcohol use and insufficient physical activity (except among men). Overall, the findings of this study suggest the need for continuous monitoring and examination of influencing factors on sub-national current tobacco use data, especially in the administrative autonomous provincial level, where some specific tobacco control policy measures can be applied, such as social marketing campaigns, smoking cessation services, effective inspection services, and comprehensive public health programs.

## Supporting Information

S1 Data Tobacco_Prevalence_APV_Serbia_2023. (XLSX)
